# Aphasia Associated With Acute on Chronic Kidney Failure in an Adolescent

**DOI:** 10.7759/cureus.49044

**Published:** 2023-11-19

**Authors:** Jacqueline A Jones, Rachel K Cason, Eileen T Chambers, Carolyn E Pizoli, Karan R Kumar

**Affiliations:** 1 Department of Pediatrics, Duke University School of Medicine, Durham, USA

**Keywords:** uremia, focal deficit, pediatric, aphemia, aphasia, kidney failure

## Abstract

Acute and chronic kidney disease (CKD) have known neurological associations resulting from uremia, electrolyte disturbances, comorbidities such as hypertension, or other toxin accumulation. Reversible focal neurological deficits are relatively uncommon and poorly understood sequelae of kidney disease. Herein, we describe an unusual case of an adolescent male who developed acute aphasia during his initial presentation for acute kidney injury (AKI) superimposed on progressive CKD stage 5 associated with uremia and multiple electrolyte derangements. Symptoms resolved within one day of initiating continuous renal replacement therapy (CRRT) and gradual electrolyte and uremia correction. Such transient focal neurological deficits in AKI superimposed on progressive CKD in the pediatric population has not been widely reported.

## Introduction

Acute and chronic kidney disease (CKD) have known associated neurological complications from various etiologies. Temporary or permanent neurological impairments can result from uremia, electrolyte disturbances, toxin accumulation, or comorbidities related to kidney diseases, including hypertensive encephalopathy, posterior reversible encephalopathy syndrome (PRES), and stroke [1,2]. The majority of neurological symptoms are nonspecific or generalized, including weakness, confusion, seizures, fatigue, nausea, headaches, disequilibrium, and muscle cramps [2,3]. However, transient focal neurological deficits are uncommonly reported symptoms of acute kidney dysfunction.

Similarly, neurological complications from kidney impairment in children are often generalized in nature or pertain the effects of chronic diseases and dialysis, such as developmental delay, buildup of aluminum toxicity, or stroke [4,5]. Little is reported on transient focal neurological deficits in the pediatric population with acute worsening of kidney failure despite observations dating to the early 1900s [6]. As such, we describe an unusual case of an adolescent male who developed acute neurological deficits including non-fluent aphasia during his initial presentation for acute kidney injury (AKI) superimposed on progressive worsening of CKD. Associated laboratory findings included hyponatremia, uremia, and multiple other electrolyte derangements. His neurologic symptoms fully resolved within one day of initiating continuous renal replacement therapy (CRRT) and gradual electrolyte and uremia correction. This presentation has not been widely reported.

## Case presentation

A 16-year-old male with no significant past medical history presented for shortness of breath for one day following two weeks of flu-like symptoms, including fever, cough, vomiting, diarrhea, and body aches, for which he was taking 800 mg ibuprofen twice daily for the past week. He reported difficulty thinking and talking the day of presentation. He was a high school athlete and had previously been in his overall normal state of health and activity. 

On examination, he was afebrile with a heart rate of 124 bpm, blood pressure 167/87, and breathing 30 respirations per minute with SpO_2_ of 100% on room air. Medical examination was notable for crackles in the lung bases, comfortable breathing, and a productive cough. While in the emergency department, he developed garbled speech and reported tongue tingling. He was initially able to complete sentences, but shortly afterward, he became unable to speak at all. He was transferred to a nearby PICU where he was noted to be alert and oriented but mute. It is unclear if he could repeat; however, he could follow commands consistent with a non-fluent aphasia with intact comprehension, but interestingly, he could also read and write readily and appropriately answer questions by texting on his phone. Pupils were equal and reactive, eye movements were normal, and face was symmetric. The only bulbar dysfunction noted was mild drooling. He had intact strength and sensation; reflexes were unremarkable. He had difficulty standing and gait instability without dysmetria, cerebellar, or sensory ataxia. 

**Figure 1:**
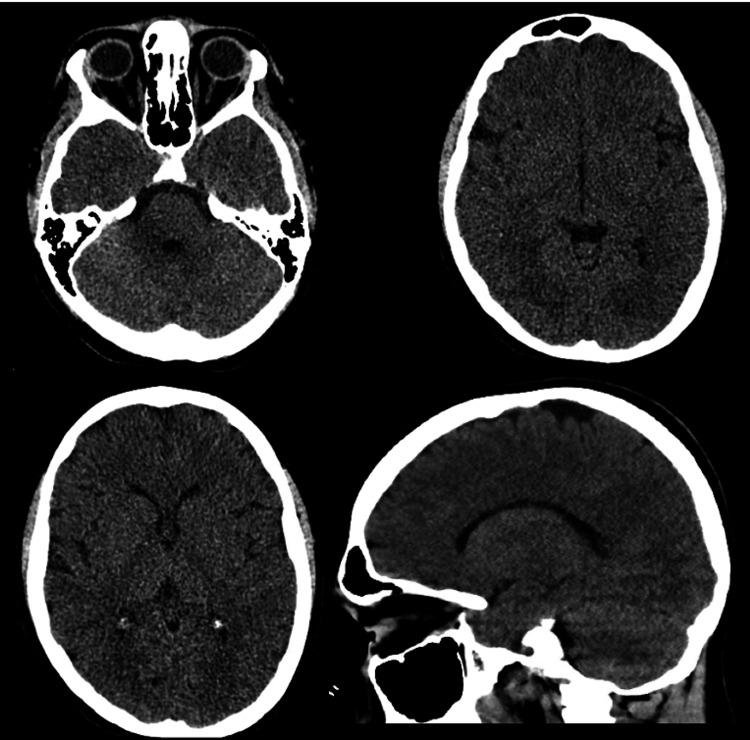
Head CT Noncontrast head CT obtained in the emergency department reported as sinusitis without acute intracranial findings.

**Figure 2:**
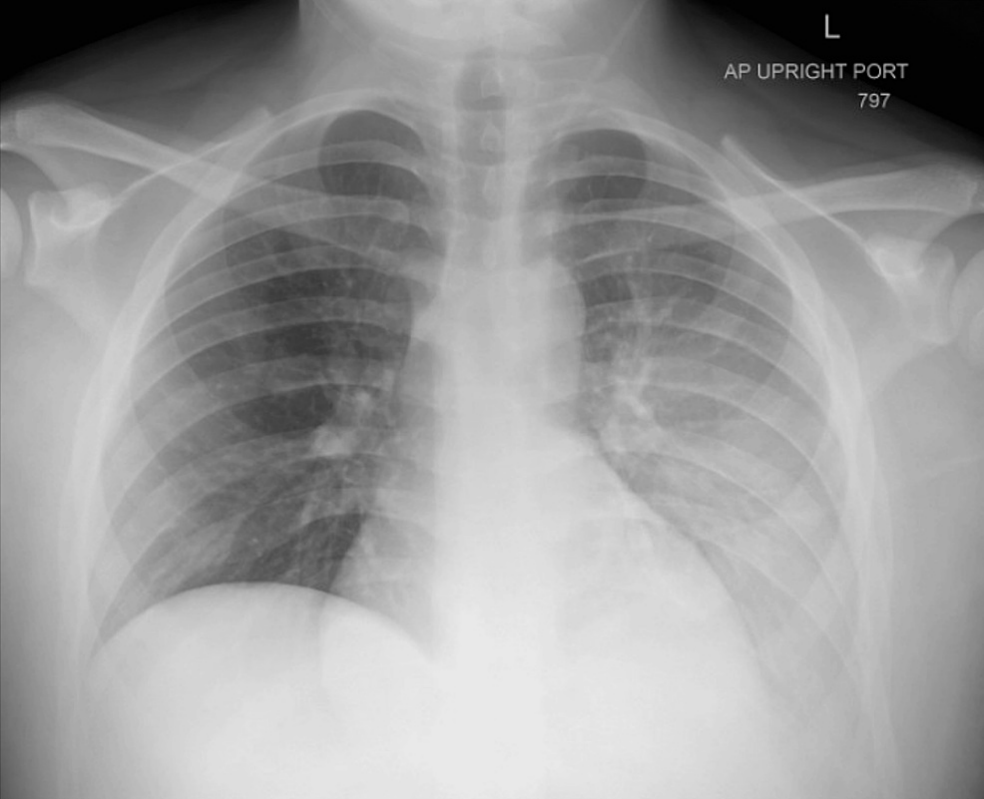
CXR CXR demonstrating a left lower lobe opacity, which could represent pneumonia or atelectasis. A small left sided effusion was later noted.

**Figure 3:**
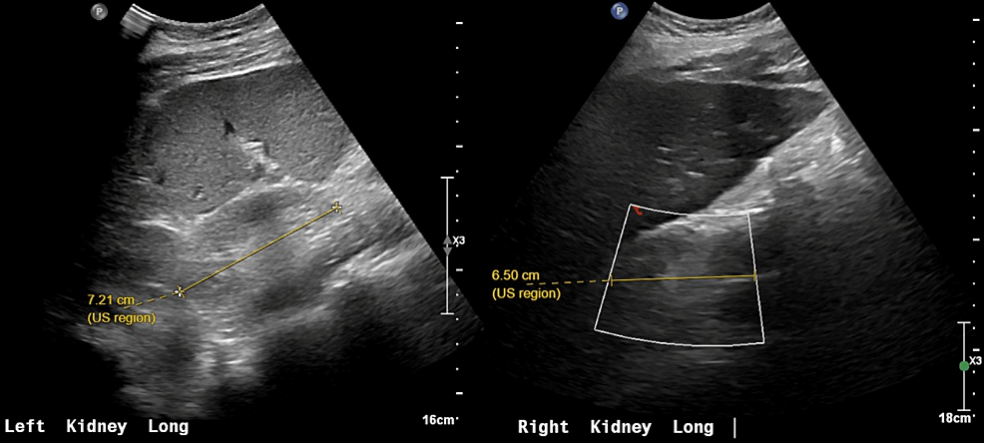
Renal ultrasound The left kidney (left) measured 7.2 cm. The right kidney (right) measured 6.5 cm, although the inferior margin was noted to be obscured by bowel gas.

Initial labs demonstrated multiple abnormalities, including severe hyponatremia (121 mmol/L), mild hyperkalemia (5.6 mmol/L), uremia (168 mg/dL), elevated creatinine (21.7 mg/dL), normocytic anemia (Hgb 6.9 g/dL), hyperparathyroidism (518 pg/mL), hypocalcemia (5.5 mg/dL), anion gap metabolic acidosis (pH 7.25), and leukocytosis. Glucose, magnesium, lactate, and liver enzymes were normal. Head CT noted sinusitis but was otherwise normal (Figure 1). CXR showed a left lower lobe opacity, later demonstrated to be a small pleural effusion (Figure 2). Renal ultrasound showed very small kidneys for age bilaterally with poor corticomedullary differentiation without masses, cysts, or calculi, or urinary tract dilation (Figure 3). Cardiac, liver, pancreas, and spleen evaluations were normal. He was diagnosed with kidney failure of unclear etiology, and CRRT was initiated that day for gradual electrolyte correction. He required intubation for dialysis catheter placement and was also started on ampicillin-sulbactam for sinusitis. On the following day, he was extubated at which time aphasia had resolved and he was back to his neurological baseline. At this time, hyponatremia (133 mmol/L), uremia (138 mg/dL), and acidosis (pH 7.40) were improved. He was discharged home on intermittent hemodialysis after approximately two weeks. Further review of past medical history revealed only a single episode of dark urine four years prior, which had been evaluated and found to be urethral scar tissue not requiring further follow-up. The etiology of his progressive CKD remains unknown despite thorough work-up ruling out cystic kidney disease, glomerulonephritis, metabolic renal diseases and other known causes of kidney failure in children. 

Duke University Health System Institutional Review Board guidelines were followed regarding IRB review and approval. Verbal consent from the patient and guardian was obtained prior to submission.

## Discussion

Here, we describe a notable transient focal neurologic deficit of expressive aphasia at the initial presentation of kidney failure in an adolescent. The patient had relative sparing of generalized symptoms despite the focality of his neurological deficits, which were not limited to a singular vascular territory. He acutely displayed a severe non-fluent aphasia with intact verbal comprehension, reading, and writing consistent with a pure word mutism or the aphemic form of “Broca’s aphasia” [7]. His cranial nerve exam was notable only for mild drooling without oro-facial-lingual weakness, making dysarthria a less likely cause. His difficulty with standing and ambulation were possibly generalized in nature given no dysmetria or sensory ataxia, and the lack of other cerebellar signs also makes cerebellar mutism unlikely. 

There are multiple potential pathophysiological mechanisms for the patient’s aphasia. The first is his electrolyte derangements, namely, the severe hyponatremia. While overwhelmingly characterized by generalized symptoms resulting from diffuse cerebral edema, severe hyponatremia is associated with focal neurological deficits in approximately 5% of cases and is a recognized rare cause of stroke mimic, including reversible, isolated Broca’s aphasia [8-11]. The mechanisms causing focal deficits in hyponatremia are poorly understood.

Another proposed mechanism for our patient’s aphasia is uremic encephalopathy. The accumulation of uremic toxins, organic acids, and development of metabolic acidosis in CKD has been linked to the development of cognitive dysfunction [12]. The metabolic acidosis may lead to increased blood-brain barrier permeability and affect local metabolism to alter cognition [12]. A study on MRI findings in 10 patients with acute or chronic renal failure and predominately motor symptoms and dysarthria demonstrated diffusion imaging consistent with vasogenic or cytotoxic edema particularly affecting the basal ganglia and cerebral cortex [13]. The results were irrespective of hypertension, electrolyte abnormalities, or acidosis, and all patients showed improvement after hemodialysis [13]. These findings remain nonspecific given the overlapping neuroimaging phenotypes seen in uremic encephalopathy elsewhere [14]. Ultimately, kidney failure may lead to the accumulation of neurotoxic compounds that cause focal and reversible disturbances. While our patient had a normal CT, he did not have an MRI performed, which may have captured more subtle findings demonstrating this effect. 

For any patient presenting with kidney failure, PRES should be considered in the differential diagnosis. PRES is a reversible neurotoxic injury of which hypertension and kidney failure are known causes. While encephalopathy and nonspecific symptoms, such as headache, seizures, and visual disturbances, are seen in up to 94% of patients, focal neurological deficits are seen in 5-15% of patients depending on the areas involved [15,16]. Symptoms develop over hours to days, and CT may show findings of vasogenic edema (although MRI is much more sensitive) [15,16]. In the present case, our patient’s parents reported he had elevated blood pressure at a clinic visit five months prior, suggesting subacute to chronic component of the hypertension. However, his head CT head was normal and he had otherwise normal mentation, which does not exclude the diagnosis but does make PRES less likely.

There are some other notable diagnoses to consider that ultimately did not appear consistent with our patient’s presentation. Drug ingestions frequently cause altered mental status. Our patient did not have a toxicology screen during his admission. However, aphasia with otherwise a normal mental status would be an unusual finding for intoxications. Another possibility is functional neurological disorder with selective mutism, but this is a diagnosis of exclusion and unlikely given the clinical context and resolution of symptoms with CRRT. Other excluded causes include metabolic derangements resulting from liver disease, hypoglycemia, and nonketotic hyperglycemic hyperosmolar states, all of which have been reported to induce focal neurological deficits, but our patient showed no evidence of these conditions [17,18].

## Conclusions

This case serves to highlight an unusual neurological finding in a pediatric patient presenting with a new diagnosis of renal failure. Reversible focal neurological impairment without global encephalopathy is an uncommon and underrecognized symptom that may present in acute and chronic kidney failure. The relative sparing of generalized symptoms is unusual and it is unclear whether this is a tendency unique to pediatrics. Furthermore, the specific focal deficit of aphemia itself is uncommon in comparison to other aphasia syndromes associated with metabolic derangements.

There are data to support that uremic encephalopathy alone can lead to reversible intracranial lesions. Transient neurotoxicity from uremia may account for the aphasia that occurred in our patient. Acidosis, hyponatremia, and PRES may also occur in this population and cause an overlap of symptoms. In our patient, it is particularly difficult to disentangle the potential effects of his hyponatremia from that of his uremia. A larger patient cohort would help to parse out the confounding effects of these various comorbidities. In addition, MRI may capture subtle brain abnormalities to provide better insights to the cause of the transient neurological symptoms. Further studies are needed to better characterize the risk factors and pathogenesis of aphasia and other focal neurological deficits occurring in pediatric patients presenting with kidney failure to better understand the direct risks of uremic toxicity while accounting for comorbidities.
